# An overview of ethnography in healthcare and medical education research

**DOI:** 10.3352/jeehp.2011.8.4

**Published:** 2011-04-25

**Authors:** Leigh Goodson, Matt Vassar

**Affiliations:** Oklahoma State University Center for Health Sciences, Tulsa, OK, USA.

**Keywords:** Ethnography, Qualitative Research, Medical Education, Healthcare

## Abstract

Research in healthcare settings and medical education has relied heavily on quantitative methods. However, there are research questions within these academic domains that may be more adequately addressed by qualitative inquiry. While there are many qualitative approaches, ethnography is one method that allows the researcher to take advantage of relative immersion in order to obtain thick description. The purpose of this article is to introduce ethnography, to describe how ethnographic methods may be utilized, to provide an overview of ethnography's use in healthcare and medical education, and to summarize some key limitations with the method.

## INTRODUCTION

Researchers from the social science disciplines are able to take advantage of a wide array of research methodologies. Such methodologies range from the traditional quantitative approaches within the positivist tradition to the qualitative approaches premised upon the ideologies of constructivism. A third alternative, mixed methods designs, have also gained popularity in recent years. Inherent to each of these approaches are different, and sometimes opposing, philosophical and epistemological views. These views dictate the nature of the research design. On one hand, positivists focus on studying that which can be directly observed and confirmed by the senses. Such research seeks to test theory-based, testable hypotheses while remaining objective and value neutral. On the other hand, constructivists believe that knowledge is socially constructed and situated within a particular context. Since each context is unique and will have different perspectives, the world has many different meanings - none of which may be more valid than another. Hence, research designs are primarily aimed at describing the context or group of interest.

Creswell [1] cites these three types of designs: qualitative, quantitative, and mixed methods. A mixed method is a combination of qualitative and quantitative. These two methods are at opposite ends of the continuum and the continuum is the level of measurement: nominal, ordinal, interval, and ratio. The type of research method used is referring to the method used to collect the data. Researchers using quantitative methods are testing theories through examining statistical relationships between variables. The data is measured numerically with an ordinal, interval, or ratio scale. Qualitative methods use a nominal scale. That is, they may use numbers to label categories but it is only for the purpose of sorting information. The number is simply a label. Qualitative methods aim to explore a culture or group of individuals to understand more about the social or human problem this group experiences. Qualitative methods are often open ended or participatory in nature. Qualitative research, whether standing alone or in a mixed method, adds rich information to any investigation otherwise not discoverable. With quantitative methods, investigators rely on literature and past surveys to target the proper information. Qualitative research allows for variable discovery. That is, the results may glean something never before addressed in similar research. The use of qualitative methods is essential to get closer to exhaustive information on any given topic or population. Oftentimes, the qualitative research leads the researcher to further quantitative investigation. These are not competing but rather complimentary methods.

Qualitative research is often overlooked as an option when considering the methodological approach to a research question. This is especially true in academic domains such as medicine where evidence-based practice has emerged as a popular treatment philosophy based largely on the quantitative research tradition. However, qualitative research can provide rich information otherwise not discovered through quantitative approaches. Ethnography is one qualitative approach that involves relative submersion into the setting to be studied, and is an appropriate methodology for a wide variety of research topics within healthcare and medical education. While, to some extent, ethnography has been applied in healthcare settings and in the medical education environment, we feel that there is a general lack of research employing this methodology. This opinion has also been expressed by Leung [2]. At times, deficits in particular research methodologies within various academic domains may be attributed to a general lack of knowledge regarding the methodology itself or ways in which the method may be applied. Therefore, the purpose of this introductory paper is to explain ethnographic methodology, discuss how the method may be used, provide a discussion of ethnography's use in healthcare and medical education, and briefly summarize some key limitations with the method. Since ethnography is a method not easily summarized in a single paper, we are writing a series of articles to follow which will address specific aspects related to conducting ethnographic research.

We begin by describing ethnography, synthesizing the works of Leung [2], Savage [3], LeCompte and Schensul [4], Pope [5], and Atkinson and Pugsley [6]. Leung [2], for example, discusses ethnography as a social research method occurring in natural settings characterized by learning the culture of the group under study and experiencing their way of life before attempting to derive explanations of their attitudes or behavior. The culturally based approach can be related to ethnicity, nationality, gender, regional origin, occupation, generation, or in healthcare the focus might be a particular pathology such as cancer, HIV, heart disease, or diabetes. Ethnographies are normally conducted in a single setting, and data collection is largely dependent upon participant observation and interviews. Savage [3] notes that ethnography may also require historical research prior to beginning actual field work. In terms of time considerations, ethnography is a research method characterized by long-term fieldwork since thick description of the participants and setting may only be acquired from sufficient exposure to them. Savage [3] further notes that ethnography is not used for developing generalized conclusions but rather studying a specific group of people regarding a specific topic and drawing conclusions only about what was studied.

According to LeCompte and Schensul [4], there are seven defining characteristics of ethnography. These include: 1) being carried out in a natural setting, not in a laboratory; 2) involving intimate, face-to-face interaction with participants; 3) presenting an accurate reflection of participant perspectives and behaviors; 4) utilizing inductive, interactive, and recursive data collection to build local cultural theories; 5) using both qualitative and quantitative data; 6) framing all human behavior within a sociopolitical and historical context; and 7) using the concept of culture as a lens through which to interpret study results.

Finally, in order to fully understand ethnographic methodology, it is necessary to briefly describe the fundamental ideas and guiding principles of ethnography which have been derived across the many academic disciplines that make use of its application. Atkinson and Pugsley [6] nicely summarize these ideas which have been succinctly stated in Table 1.

## USES OF ETHNOGRAPHY

Ethnography is a useful qualitative approach to address a particular type of research question. LeCompte and Schensul [4] suggest that ethnography should be used to:

- Define a problem when the problem is not yet clear.
- Define a problem when it is complex and embedded in multiple systems or sectors.
- Indentify participants when the participants, population sectors, stakeholders, or the boundaries of the study population are not yet known or identified.
- Clarify the range of settings where a problem or situation currently occurs when not all of the possible settings are fully identified, known, or understood.
- Explore the factors associated with a problem in order to indentify, understand, and address them either though research or intervention studies, when they are not known.
- Document a process.
- Identify and describe unexpected or unanticipated outcomes.
- Design measures that match the characteristics of the target population, clients, or community participants when existing measures are not a good fit or need to be adapted.
- Answer questions that cannot be addressed with other methods or approaches.
- Ease the access of clients to the research process and its products.

Consider the following example by Perry et al. [7] on the use of ethnography to address a research question.

Summary: Many individuals must deal with loved ones who are too disabled to care for themselves. As pressure continues to keep healthcare costs down, many patients stay fewer days in the hospital after an injury or illness. Perry et al [7]. described the challenges associated with releasing patients from the hospital before they are fully recovered. Nurses often become the supervisor of the care rather than the primary caregiver. This trend brings to light potential conflicts between supervisor and caregiver. Perry et al. acknowledge the many nursing scholars who contend we must see culture as part of the patient plan. In response to these issues, Perry et al. used ethnography to examine the process of resisting vulnerability as it relates to health care professionals and health system structure. The purpose of the Perry study was to describe the family's view when a family member is in the hospital. The research team concluded families of diverse backgrounds needed to work harder to resist vulnerability in the healthcare system.

Method use: Perry et al. used feminist ethnography methods. This ethnographic method begins with the individual's experiences and broadens their view from there to better understand the decision making process the families of non-Caucasian backgrounds. Two studies were conducted: one for the in-hospital experience and the second after the patient was discharged.

Data: In ethnography, data collection can sometimes change along the course of the study. A conversation with one study participant may lead to generation of further questions. This happened in the Perry study. Both family members and healthcare providers were interviewed for this study. Once data was collected, it was then coded, and reviewed for accurate interpretation.

Report: The management of vulnerability by the family was the focus of this study. Families felt pulled toward vulnerability when they perceived a lack of two way communication from the healthcare providers. When families felt heard by the healthcare providers, they felt a resistance to vulnerability. With communication as a focal point in the vulnerability of the patient, language was identified as important to the perception of the families. Perry et al. emphasized the recognition of cultural and linguistic differences when working with families to care for the patient.

In this case, ethnography was a useful method in addressing the research question. In order to fully understand the family's perspective regarding premature hospital release as well as the responsibility of such caregiving, the researchers needed to understand the complex nature of these phenomena. Ethnography was chosen since it allows for thick description of these underlying issues.

In general, a deep understanding to a research question is nicely achieved through the use of ethnography. Rice and Ezzy [8] contend that there are several benefits to conducting an ethnography: diverse cultures are better understood through a deep understanding of why people behave in certain ways and have certain beliefs, it is a strategy for the development of grounded theory, and it best addresses the needs of humans.

## ETHNOGRAPHIC STUDIES IN HEALTHCARE AND MEDICAL EDUCATION

While cultural understanding is the most cited use of ethnography, there are many specific settings where the method is quite valuable. One of those areas is healthcare. The vast numbers of variables in the clinical setting lend themselves well to be analyzed in a more open ended approach rather than answering questions from a survey or pulling archived data from the hospital and clinic databases [8]. The patient/nurse relationship is the key to the success of healthcare. Better understanding those dynamics allows decision makers to proceed with more reliable and pertinent information. Getting to the root issues about patient care rather than tracking behavior leads to real solutions rather than a trial and error process.

Campbell [9] also suggests the use of qualitative research in health care. A large field in itself, health policy would benefit from the complimentary methods of qualitative analysis. Further suggested was meta-ethnography as a qualitative compliment to quantitative meta-analysis [9]. Through a study involving diabetic care, Campbell [9] concluded a meta-ethnography was possible and advantageous. The issue of non-compliance was studied with the benefit of seven different studies. Campbell [9] further suggested methodology within the studies included needed to be better documented a suggestion for further development of meta-ethnography.

Garro [10] nicely summarizes the uses of ethnography in health care decisions. It begins with the most basic of decisions as to whether or not to seek health care. Garro [10] goes on to cite several examples of use in medicine. Women deciding to go to the hospital to have their baby generally go too early, hence requiring them to go home and come back again. The Scrimshaw and Souza study cited by Garro [10] goes on to describe a dynamic where healthcare providers assumed patients understood the instructions the same way as the healthcare provider. The result and further action item was to redesign instructions in two different languages to accommodate the different interpretive tendencies of the patients. Cultural factors continue to be a major factor in the delivery of healthcare. Patients make decisions through this lens creating a dynamic in the healthcare system with a wide variance of options for the various cultures served.

Garro [10] finally concludes her review with an example leading to an interesting conclusion. Two Mexican towns were studied to determine decision making patterns as they relate to healthcare. The towns were thought to have similar beliefs and the interviewing techniques for these two towns were different. Again here, we see ethnography can be tailored to the population being studied. The flexibility is very useful. The two techniques yielded similar results in terms of the medical knowledge of the two communities. The difference in the two communities was access. The town having greater access to healthcare sought medical attention at twice the rate of the town lacking access. The Garro study indicated access to healthcare was more indicative than illness beliefs when determining how and when a patient sought care [10].

Van der Geest and Finkler [11] suggest the use of ethnography in the hospital setting. Hospitals are often cultures within themselves. And, while some can be very similar, the community of the hospital is often unique. Because hospitals reflect dominant culture and belief systems, the care in each hospital can be different based on the cultural influences. This is not clear to the naked eye. From the outside, hospitals look and operate similarly. The patient care and decision making processes can vary widely. Rice and Ezzy [8] suggest that, through ethnography, behaviors are understood and used to treat the patient through means that fit the needs of the patient. The benefits brought by the ethnography are understanding of the social and cultural backgrounds of the patients and how health behaviors differ across groups. Savage [3] cites useful ethnographies in health care looking at various issues from cultural differences among clinic attendees to the clinical reasoning differences among physician specialists.

Ethnography has been used in medical education for more than 50 years. Two landmark studies conducted in the United States show earlier uses of ethnography. The first, entitled *The Student Physician*, was a collection of research from several medical schools. Within this collection, Fox's ethnographic project on medical innovations in clinical settings or the enthographic account of uncertainty in medical knowledge has influenced much subsequent research [6,12].

*Boys in White*, the second ethnographic account, focused on medical student culture. In Atkinson and Pugsley's [6] account, this study drew "an explicit parallel between student cultures in universities and shop-floor cultures in workplace settings. Workers and students alike established shared perspectives on their shared problems and collective responses to shared demands" (p. 233). Medical students were found to more effectively manage these demands by using selective negligence (only learning vital information). More recent uses of ethnography in medical education have been noted and will be discussed in our series of subsequent articles as exemplars of ethnographic research.

## LIMITATIONS OF ETHNOGRAPHY

Sample size is a limitation of the ethnography method. The time required being involved in participant observation and conducting long interviews greatly limits the sample size. Unlike a scan sheet used for a common survey, ethnography is laborious and detailed in the collection of data.

It is difficult to generalize with the ethnography method. When researching a certain culture, the results cannot necessarily be generalized to other populations. Because the results are based on the cultural responses, the outcome of the study cannot be applied beyond where the study was conducted. For instance, if a best practice ethnography was conducted for the Emergency Room in a hospital, the same best practices may not be applied to the hospital on the other side of town. The best practices for hospital number one were developed based on the population using that hospital and the administration running that hospital.

Subjectivity is certainly a limitation of ethnography. The interpretation of the cultural experience will vary among researchers. There is not a list of answers from which to choose but rather the use of notes made by the investigator and later interpreted and categorized by the investigator. The entire project is subject to the processes and interpretations developed by the researcher and the research team. Not so with quantitative research. With quantitative, there is a measurable response from which to draw conclusions. It is objective.

Funders are often reluctant to fund such projects. Some feel this type of research can lack generalisability [3]. Another issue for funders is the great expense. It is much less expensive to conduct research with a survey than to immerse an investigator into a culture for a given period of time to extract extensive information.

Finally, the biggest challenge an ethnographer may encounter is the acceptance of the culture. Should the researcher choose ethnography, they must be accepted temporarily within the culture in order to gather accurate information. Hence the need to conduct thorough reviews prior to beginning the ethnography. Additionally, the development of key informants is essential.

## CONCLUSION

Qualitative research, while time consuming, is an excellent tool to investigate differences among cultures, genders, professions, and geographic regions, to name a few. It provides a rich collection of information for the investigator. Oftentimes, it is the source of determining variables for further research and can therefore serve as the launching pad for larger original studies. While we have seen some use of qualitative research within healthcare and medical education, it is underrepresented compared to its quantitative counterpart. Of these qualitative approaches, ethnography is a method quite amenable to medicine, and the application of ethnography to healthcare is widely supported. Health behaviors and differences in healthcare delivery are not necessarily detected in a quantitative study. The use of ethnography allows the decision maker to have a better understanding of the patient and the healthcare delivery team.

## Figures and Tables

**Table 1:**
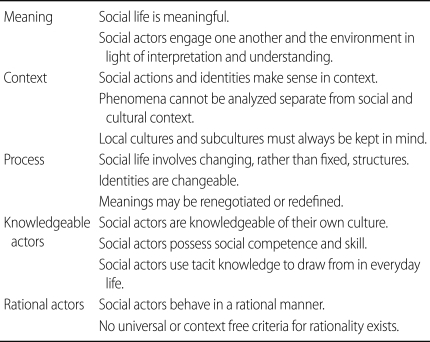
Fundamental ideas underlying ethnography
